# SUMOylation of Grb2 enhances the ERK activity by increasing its binding with Sos1

**DOI:** 10.1186/1476-4598-13-95

**Published:** 2014-04-29

**Authors:** Yingying Qu, Qin Chen, Xueping Lai, Changhong Zhu, Cheng Chen, Xian Zhao, Rong Deng, Ming Xu, Haihua Yuan, Yanli Wang, Jianxiu Yu, Jian Huang

**Affiliations:** 1 Department of Biochemistry and Molecular Cell Biology, Shanghai Key Laboratory of Tumor Microenvironment and Inflammation, Shanghai Jiao Tong University School of Medicine (SJTU-SM), Shanghai 200025, China; 2 State Key Laboratory of Oncogenes and Related Genes, Shanghai Jiao Tong University School of Medicine, Shanghai, China; 3 Department of Oncology, No. 3 People’s Hospital Affiliated to Shanghai Jiao Tong University School of Medicine, Shanghai, China

**Keywords:** Grb2, SUMOylation, Sos1, ERK activity, Tumorigenesis, Cell migration

## Abstract

**Background:**

Grb2 (Growth factor receptor-bound protein 2) is a key adaptor protein in maintaining the ERK activity *via* linking Sos1 (Son of sevenless homolog 1) or other proteins to activated RTKs, such as EGFR. Currently, little knowledge is available concerning the post-translational modification (PTM) of Grb2 except for its phosphorylation. Since emerging evidences have highlighted the importance of SUMOylation (Small ubiquitin-related modifier), a reversible PTM, in modulating protein functions, we wondered if Grb2 could be SUMOylated and thereby influences its functions especially involved in the Ras/MEK/ERK pathway.

**Methods:**

SUMOylation of Grb2 was analyzed with the *in vivo* SUMOylation assay using the Ni^2+^-NTA affinity pulldown and the *in vitro E.coli*-based SUMOylation assay. To test the ERK activity and cell transformation, the murine fibroblast cell line NIH/3T3 and the murine colon cancer cell line CMT-93 were used for the experiments including Grb2 knockdown, ectopic re-expression, cell transformation and migration. Immunoprecipitation (IP) was employed for seeking proteins that interact with SUMO modified Grb2. Xenograft tumor model in mice was conducted to verify that Grb2 SUMOylation regulated tumorigenesis *in vivo*.

**Results:**

Grb2 can be SUMOylated by SUMO1 at lysine 56 (K^56^), which is located in the linker region between the N-terminal SH3 domain and the SH2 domain. Knockdown of Grb2 reduced the ERK activity and suppressed cell motility and tumorigenesis *in vitro* and *in vivo*, which were all rescued by stable ectopic re-expression of wild-type Grb2 but not the mutant Grb2^K56R^. Furthermore, Grb2 SUMOylation at K^56^ increased the formation of Grb2-Sos1 complex, which sequentially leads to the activation of Ras/MEK/MAPK pathway.

**Conclusions:**

Our results provide evidences that Grb2 is SUMOylated *in vivo* and this modification enhances ERK activities *via* increasing the formation of Grb2-Sos1 complex, and may consequently promote cell motility, transformation and tumorigenesis.

## Background

SUMOylation is a post-translational modification featured by covalent and reversible attachment of small ubiquitin-like modifier (SUMO) to protein substrates at specific lysine residues [1,2]. SUMOylation has emerged as an important regulatory mechanism in many eukaryotic cell processes and physiological events including sub-cellular localization, transcription activation, DNA synthesis and repair, cell cycle regulation and chromatin organization [3-5], which are all involved in human disease pathogenesis including tumorigensis [6].

Growth factor receptor-bound 2 (Grb2) is a ubiquitously expressed adaptor protein involved in several tyrosine-kinase dependent signaling pathways [7]. Grb2 contains a structure with one Src homology 2 (SH2) domain flanked by two SH3 domains [8]. The SH2 domain helps Grb2 interact directly with phosphorylated tyrosine residues of receptor tyrosine kinases (e.g., EGFR, PDGFR) and non-receptor tyrosine kinases (e.g., FAK, Bcr/Abl), while the SH3 domains mainly bind to proteins containing proline-rich motifs, such as Sos1 [9]. As a pivotal signal adaptor protein, Grb2 contributes to cell proliferation and normal development by linking other proteins to the membrane after the recruitment of activated EGFR. Upon the activation of EGFR or other RTKs, Grb2 recruits Sos1 to the membrane to form Grb2-Sos1 complex, which is crucial for signaling transduction and sequentially leads to the activation of Ras/MEK/MAPK (generally recognized as extracellular signal-regulated kinase, ERK) [10].

In the last two decades, a major focus of Grb2 research is to identify its new binding proteins and the roles involved in classical or unconventional signaling pathways. However, except for phosphorylations [11], other post-translational modifications (PTM) of Grb2 and their functions remain to be explored. In this study, we found that Grb2 could be SUMOylated at K^56^, which facilitated the binding of Grb2 to Sos1 and subsequently enhanced the ERK activity. Our study reveals a new regulatory mechanism for Grb2-controlled ERK activity *via* SUMO-dependent Grb2-Sos1 complex formation.

## Results

### Grb2 is SUMOylated *in vivo* and *in vitro*

**Figure 1:**
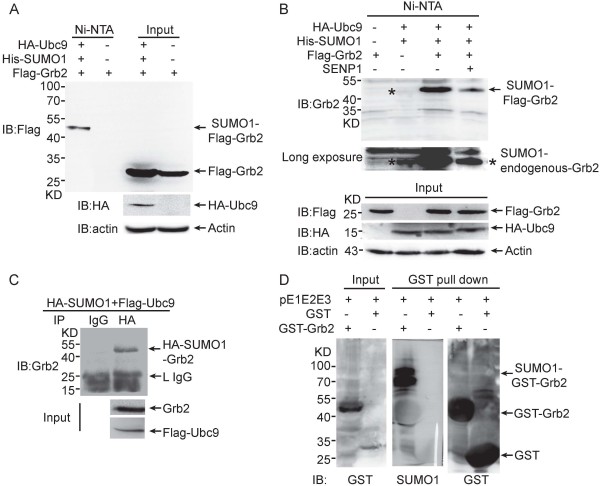
**Grb2 is SUMOylated *in vivo *and *in vitro*. (A)** HEK293T cells were transfected with Flag-Grb2 along with or without His-SUMO1 and HA-Ubc9. SUMOylated proteins were purified from cell lysates using Ni^2+^-NTA affinity pulldown (Ni-pulldown) and SUMOylated Grb2 was detected by immunoblotting for the Flag tag. **(B)** His-SUMO1-conjugated proteins were purified by Ni-pulldown from HEK293T cells expressing Flag-Grb2, His-SUMO1, HA-Ubc9 or SENP1 and probed for Grb2. *indicates endogenous SUMOylated Grb2. **(C)** HEK293T cells were co-transfected with HA-SUMO1 and Flag-Ubc9. Lysates were immunoprecipitated with anti-HA(m) antibody or mouse IgG (as a control), and then immunoblotted with anti-Grb2 antibody. **(D)** The pE1E2S1 plasmid was co-transformed with pGEX4T1 (GST) or pGEX4T1-Grb2 (GST-Grb2) into *E.coli* BL21(DE3). Proteins were purified with GST agarose beads followed by Western blotting analysis.

To investigate whether Grb2 could be SUMOylated *in vivo*, we transiently transfected Flag-tagged Grb2 along with His-tagged SUMO1 and HA-tagged SUMO E2 ligase Ubc9 in HEK293T cells. Following the purification of His-tagged SUMO1 conjugates with Ni^2+^-NTA agarose beads as described in our previous studies [12,13], Grb2 was detected with anti-Flag antibody in the cells co-transfected with Ubc9 and SUMO1 but not in the cells transfected with Flag-Grb2 alone (Figure 1A). Upon the covalent linkage of SUMO1 to Grb2, the relative molecular mass of Flag-Grb2 shifted from 26 to 46 kDa. Moreover, the SUMOylation of Grb2 was massively weakened upon co-transfection of the SUMO deconjugase SENP1 (Figure 1B). Additionally, long exposure of the membrane incubated with anti-Grb2 antibody showed that endogenous Grb2 was also SUMOylated (Figure 1B, lane 2 in the second panel). To further confirm endogenous Grb2 is SUMOylated, HEK293T cells were transfected with HA-SUMO1 and Flag-Ubc9, and then the lysates were used for immunoprecipitation (IP). Convincingly, as shown in Figure 1C, the SUMO1-Grb2 band was clearly detected by anti-Grb2 antibody when the lysates were immunoprecipitated with anti-HA antibody but not with anti-normal IgG.

We also examined the SUMOylation of Grb2 using an *in vitro E.coli*-based SUMOylation assay by pE1E2S1 [14]. pE1E2S1 contains the linear fusion of Aos1/Uba2, Ubc9 and SUMO1 gene coding sequences which can express in *E.coli* strain BL21(DE3) and modify the potential substrate proteins with SUMO1. As shown in Figure 1D, the SUMO1-GST-Grb2 with higher molecular weight of approximately 70 kDa was detected in the *E.coli* transformed with pE1E2S1 and GST-Grb2 (with an expected size of 50 kDa) but not in the *E.coli* transformed with pE1E2S1 and GST vector. Collectively, these data demonstrated that Grb2 is a SUMOylated protein.

### K^56^ is the major site for Grb2 SUMOylation

**Figure 2:**
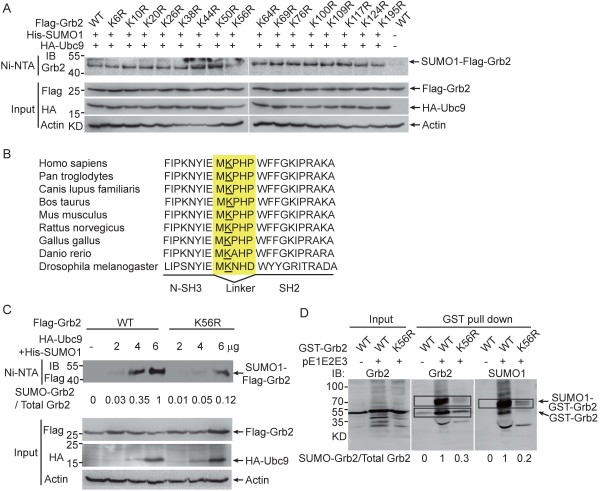
**K**^**56 **^**is a critical site for Grb2 SUMOylation. (A)** HEK293T cells were co-transfected with Flag-Grb2 wild-type or mutants along with or without His-SUMO1 and HA-Ubc9. The *in vivo* SUMOylation assay using Ni^2+^-NTA beads was conducted as described in Methods. **(B)** Amino acid sequence alignment of Grb2 partial sequences located between N-SH3 and SH2 domains from different species. The conserved lysine in the linker region (yellow) has been underlined. **(C)** HEK293T cells were transfected with Flag-Grb2^WT^ or Flag-Grb2^K56R^ plasmid along with the increased amount of His-SUMO1 and HA-Ubc9 plasmids. The levels of SUMOylation were determined as described in Methods. **(D)** The plasmid GST-Grb2 or GST-Grb2^K56R^ was co-transformed with pE1E2S1 plasmid into BL21(DE3). Western blotting was conducted after GST pulldown.

To identify the critical site(s) for Grb2 SUMOylation, we analysed Grb2 mutants in which all 16 lysines were mutated individually to arginines for SUMOylation identification. As shown in Figure 2A, only the mutation of Lys-56 (K^56^) significantly decreased the level of SUMOylated Flag-Grb2, suggesting that Grb2 is mainly SUMOylated at K^56^. Interestingly, K^56^ is located in the linker region between the N-SH3 domain and the SH2 domain, and K^56^ and its flanking sequences are highly conserved among vertebrate and invertebrate (Figure 2B). We further compared the degree of SUMOylation between Grb2^WT^ and Grb2^K56R^ by increasing the amount of Ubc9 and SUMO1 plasmids. As shown in Figure 2C, the SUMOylation of both Grb2^WT^ and Grb2^K56R^ were difficult to be detected in the cells co-transfected with 2 μg of Ubc9 and SUMO1, whereas SUMOylation of Grb2^K56R^ was still very low and only about 10-12% modification compared to that of Grb2^WT^ in the cells co-transfected with 6 μg of Ubc9 and SUMO1. To further confirm K^56^ is a major SUMOylation site for Grb2, we also generated GST-tagged Grb2^WT^ and Grb2^K56R^ for the *in vitro E.coli*-based SUMOylation assay. The result showed that SUMOylation of GST-Grb2^K56R^ was reduced by 70-80% compared to that of Grb2^WT^ (Figure 2D). It should be noted that there is still measurable SUMO-Grb2 when K56 was mutated, suggesting the possible existence of complementary/additional SUMO sites.

### SUMOylation of Grb2 is crucial for cellular transformation

**Figure 3:**
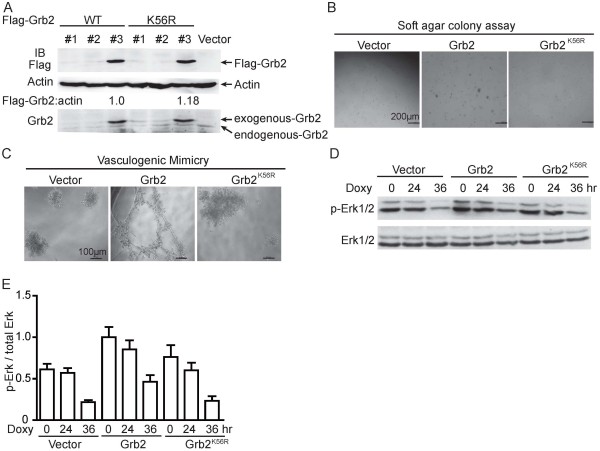
**SUMOylation of Grb2 at K**^**56 **^**promotes cellular transformation. (A)** Comparable expression levels of Grb2^WT^ and Grb2^K56R^ in stable NIH/3T3 cell lines generated by polyclonal lentiviral infections were determined by Western blotting. **(B)** Stable NIH/3T3 cell lines were seeded in 2 ml of medium containing 5% FBS with 0.35% agar at 5 × 10^4^ cells/well and layered onto the base. The photographs were taken 3 weeks later. **(C)** Vasculogenic mimicry assay for stable NIH/3T3 cell lines. Each cell line at a density of 1 × 10^4^ was plated in each well which was coated with matrigel and photographs were taken with microscope 20 hours later. **(D)** Stable NIH/3T3 cell lines at 60% confluence were stimulated with 5 μg/ml of Doxycycline (Doxy) for 24 or 36 hours. The ERK activities were determined by Western blotting. **(E)** Densitometric readings were obtained with ImageJ V1.45 software, and the signals of phosphorylated Erk were normalized to that of total Erk. Data are representative of three independent experiments.

Given that Grb2 acts as an oncogene in most mammalian cancer development [15], we asked whether SUMOylation of Grb2 is involved in the cellular transformation. NIH/3T3 stable cell lines were generated by polyclonal lentiviral infections with the Lenti-Vector carrying Grb2^WT^ or Grb2^K56R^. Two cell clones with the comparable expression levels of Grb2^WT^ and Grb2^K56R^ were chosen for the following experiments (Figure 3A).

To determine whether Grb2 SUMOylation affects the transforming potential, a soft agar colony-forming assay was carried out. As shown in Figure 3B, the colonies from the Grb2^WT^ transfectants were markedly larger than that from the Vector transfectants. However, the Grb2^K56R^ transfectants produced numbers and sizes of colonies equivalent to those produced by the Vector transfectants, indicating that Grb2^K56R^ lost its oncogenic function in promoting anchorage-independent growth (Figure 3B). Vasculogenic mimicry (VM) usually refers to the plasticity of aggressive cancer cells forming *de novo* vascular networks [16] and VM formation can be used to assess cellular transformation, so next we used the VM assay to confirm the effect of Grb2 SUMOylaiton on the transformation potential. Consistently, over-expression of Grb2^WT^ strongly induced formation of vascular-like shape whereas Grb2^K56R^ did not (Figure 3C).

Since the above results suggested that Grb2 SUMOylation at K^56^ is essential for its function in regulation of cell transformation, we wondered which signaling pathways (such as ERK, AKT, STAT3 pathways) were affected by Grb2 SUMOylation. Stable NIH/3T3 cell lines were treated with 5 μg/ml of Doxycycline for 24 or 36 hours as reported [17,18]. Grb2^K56R^ cells were incapable of maintaining the ERK activity while Grb2^WT^ cells still kept higher ERK activity with the treatment of Doxycycline for 24 h (Figure 3D & E). However, pAKT and pSTAT3 showed no much change among these cell lines (data not shown). Taken together, these data demonstrated that SUMOylation of Grb2 at K^56^ is required for up-regulation of ERK activation, which is crucial for cellular transformation.

### SUMOylation of Grb2 promotes migration and tumorigenesis *via* upregulation of the ERK activities

**Figure 4:**
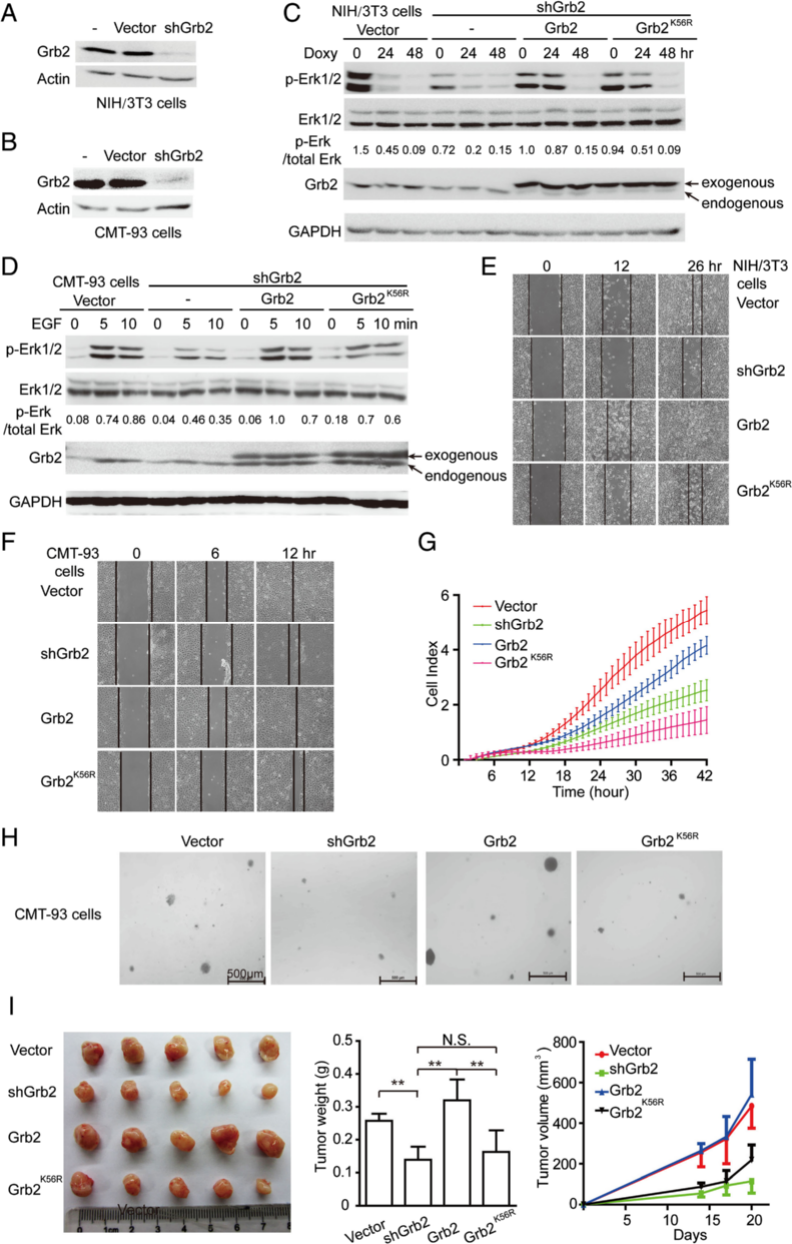
**SUMOylation of Grb2 promotes migration and tumorigenesis by upregulation of the ERK activities. (A-B)** Endogenous Grb2 in the murine fibroblast cell line NIH/3T3 (A) and the murine colon cancer cell line CMT-93 **(B)** was knockdown by a short hairpin RNA targeting Grb2 3′UTR (shGrb2) by using the lentiviral vector pLKO.1 system. Grb2^WT^ and Grb2^K56R^ were reintroduced respectively into these stable Grb2-silencing NIH/3T3 and CMT-93 cells by the lentiviral system. **(C)** The ERK activities in stable NIH/3T3 cell lines treated with 5 μg/ml of Doxycycline for 24 or 48 hours were determined by Western blotting. **(D)** Serum-starved stable CMT-93 cell lines were stimulated with 100 ng/ml of EGF for 5 or 10 minutes and the ERK activities were determined by Western blotting. **(E-F)** Cell motility was determined by wound healing assay in μ-Dish for NIH/3T3 **(E)** and CMT-93** (F)** stable cell lines. **(G)** The kinetic information about the migration of CMT-93 stable cell lines was recorded using the x CELLigence RTCA-DP system. **(H)** Stable CMT-93 cell lines were seeded in 2 ml of medium containing 20% FBS with 0.35% agar at 5 × 10^3^ cells/well and layered onto the base. The photographs were taken 2 weeks later. Images are representative of three independent experiments. **(I)** Stable CMT-93 cell lines (2 × 10^6^) were injected subcutaneously into male BALB/c nude mice (n = 5) individually. The sizes of tumors were measured at day 14, 17 and 20 after injection and the tumors were weighed.

To verify the Grb2 dependence of the ERK activities and oncogenic phenotypes, endogenous Grb2 was silenced (Figure 4A-B) before Grb2^WT^ and Grb2^K56R^ were reintroduced respectively in the murine fibroblast cell line NIH/3T3 (Figure 4C) and the murine colon cancer cell line CMT-93 (Figure 4D) using the lentiviral vector system. Then we tested the ERK activity after NIH/3T3 stable cell lines were treated with Doxycyline and CMT-93 stable cell lines treated with EGF. As expected, ERK activities were markedly reduced by Grb2 knock-down in both NIH/3T3 and CMT-93 cells. Ectopic re-expression of Grb2^WT^ in NIH/3T3 cells retained higher ERK activities compared to those of Grb2^K56R^ after treatment with Doxycycline for 24 h (Figure 4C). Similarly, Grb2^K56R^ reduced EGF-induced phosphorylation of ERK in CMT-93 cells (Figure 4D). These data further confirmed that blockage of SUMOylation of Grb2 at K^56^ impairs Grb2-regulated ERK activities.

To investigate whether the enhanced ERK activities mediated by Grb2 SUMOylation also influences cell migration, wound healing assay [19] and RTCA (real-time cell analysis) assay [20] were carried out to evaluate cell motility. As reported previously [21], knock-down of Grb2 impaired cell motility, which is consistent with the decrease of the ERK activities in Grb2-silencing NIH/3T3 or CMT-93 cells. Furthermore, ectopic re-expression of Grb2^WT^ but not Grb2^K56R^ in the endogenous Grb2-silencing NIH/3T3 or CMT-93 cells recovered the impaired cell migratory behavior (Figure 4E-G). We also determined whether the ERK activities controlled by Grb2 SUMOylation were associated with tumorigenesis in CMT-93 cancer cells. As shown in Figure 4H and Additional file 1, knockdown of endogenous Grb2 effectively suppressed the size of colonies, which was rescued by ectopic re-expression of Grb2^WT^ but not Grb2^K56R^. In order to verify the role of Grb2 SUMOylation in tumorigenesis *in vivo*, the four CMT-93 cell lines were injected subcutaneously into nude mice. Consistent with the results of colony formation experiment, knockdown of endogenous Grb2 dramatically suppressed tumor growth, which was rescued by ectopic re-expression of Grb2^WT^ but not Grb2^K56R^ (Figure 4I). Thus, our data revealed that Grb2 SUMOylation is required for promoting migration and tumorigenesis by up-regulation of the ERK activities.

### Grb2 SUMOylation increases its binding to Sos1

Our above results have proven the new concept that Grb2 SUMOylation is crucial for maintaining the ERK activities, sequentially resulting in cellular transformation, enhanced migration and tumorigenesis, so next we want to get insight into the underlying molecular mechanisms. One possibility is that Grb2 SUMOylation affects its binding with some important proteins that are closely related with the activation of the RAS/MEK/MAPK signaling pathway. To verify this hypothesis, we selected 6 target proteins including EGFR, SHP2, FAK, Sos1, PTPα and Gab1, which are all considered controlling the ERK activities and cell phenotypes (Additional file 2). EGFR was one of the most important RTKs in recruiting Grb2-Sos1 complex to regulate ERK activity in response to extracellular EGF stimulation [8,22]. Sos1, as described before, was the famous guanine nucleotide-exchange factors in directing exchange of Ras-GDP to Ras-GTP to activate ERK by binding to Grb2 SH3 domains [23]. SHP2 (Src Homology 2 containing protein tyrosine phosphatase 2) is an important tyrosine phosphatase involved in various pathophysiological processes [24] through binding with Grb2 to regulate the ERK, Akt, STAT3 signaling pathways [25-27]. Gab1 (Grb2-associated-Binding protein 1) binds to Grb2 C-SH3 domain regulating the ERK and PI3K/Akt signaling pathways in the presence of different signal factors [28,29]. The phosphorylated FAK (Focal Adhesion Kinase) binds to Grb2 SH2 domain in the help with Src regulating ERK signaling pathway and especially cell mobility [30]. PTPα (Receptor protein-tyrosine phosphatase α) also binds to Grb2 to regulate the ERK activity and tumorigenesis [31-34].

**Figure 5:**
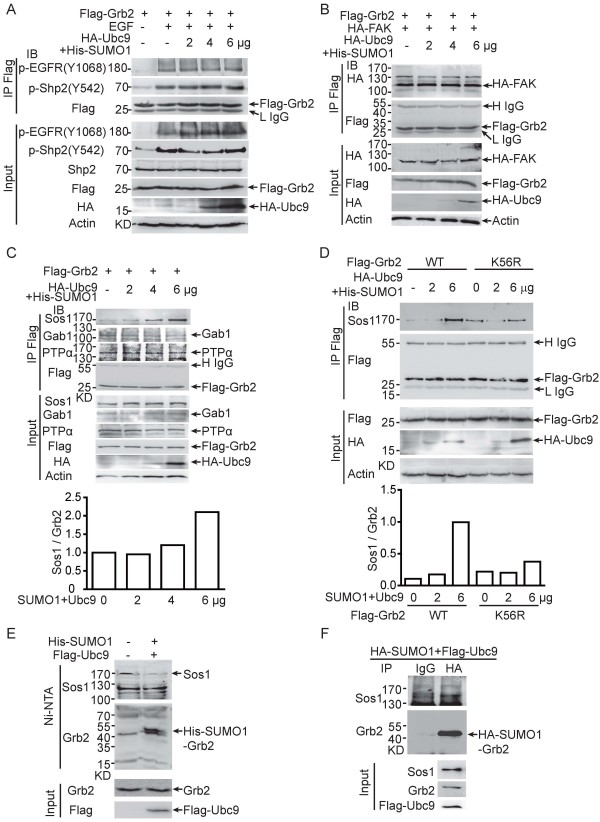
**SUMOylation of Grb2 increases its binding with Sos1. (A-B)** HEK293T cells were co-transfected with the indicated plasmids. 24 hours later, cells were subjected to serum deprivation for 20 hours, followed the treatments with or without 100 ng/ml of EGF **(A)** or 10% serum **(B)** for the indicated time. Cell lysates were immunoprecipitated and subsequently immunoblotted with indicated antibodies. **(C-D)** HEK293T cells were co-transfected with the indicated plasmids and harvested after 48 hours. Cell lysates were immunoprecipitated and subsequently immunoblotted with the indicated antibodies. Densitometric readings were obtained with ImageJ V1.45 software. **(E-F)** Sos1 was not SUMOylated. HEK293T cells were co-transfected with the indicated plasmids and analyzed for SUMOylation by Ni^2+^-NTA pulldown **(E)** or immunoprecipitation **(F)**. SUMOylated Grb2 was used as a positive control of SUMOylation, and mouse IgG as a negative control in immunoprecipitation.

The degree of Grb2 SUMOylation was gradually enhanced with the increase of the amount of SUMO1/Ubc9 plasmids in HEK293T transfection system (Figure 2C). Thus, we took advantage of gradually increasing the amount of SUMO1/Ubc9 along with equal amount of Flag-Grb2 to observe the changes in binding between SUMO-Grb2 and interacting proteins. Flag antibody was used for co-immunoprecipitation of Flag-Grb2 and the following Western blotting was conducted to detect its interacting proteins. Firstly, we questioned if Grb2 SUMOylation could influence its SH2 domain binding with EGFR and SHP2. Before harvested, transfected HEK293T cells were serum-starved for 20 h and then stimulated with EGF for 5 min (Figure 5A) or serum for 20 min (Figure 5B). As shown in Figure 5A-B, p-EGFR, p-SHP2 and FAK displayed no changes in binding with Grb2 with the increased amount of SUMO1/Ubc9 plasmids. Surprisingly, we observed that Sos1 bound to Grb2 was gradually increased whereas PTPα or Gab1 bound to Grb2 was not changed with the increase of the amount of SUMO1/Ubc9 plasmids (Figure 5C). To further validate this finding, we examined the effect of the SUMO-site mutant Flag-Grb2^K56R^ on the binding with Sos1. Indeed, Grb2^K56R^ did not show an evidently increased binding capability with Sos1 when the amount of SUMO1/Ubc9 plasmids increased (Figure 5D). Moreover, to exclude the possibility that SUMOylation enhancing the formation of Grb2-Sos1 complex is attributed to Sos1 SUMOylation, we investigated whether endogenous Sos1 can be SUMOylated by using *in vivo* Ni^2+^-NTA pulldown (Figure 5E) and immunoprecipitation (Figure 5F) assays. As shown in Figure 5E-F, endogenous Sos1 was not SUMOylated while endogenous Grb2 SUMOylation occurred as positive controls here. Taken together, our results demonstrated that Grb2 SUMOylation increases its binding to Sos1, thereby leading to the activation of the RAS/MEK/MAPK signaling pathway.

## Discussion

Grb2, containing one SH2 and two SH3 domains, works as a powerful adaptor protein by linking hundreds of adaptive signal factors to activated EGFR, which drive them to locate on the membrane. Initially, Grb2 was characterized to participate in cell proliferation, however, the recent reports indicated that Grb2 also plays a role in tumorigenesis and cell motility [15], which are consistent with our concept that Grb2 SUMOylation promotes cell migration and tumorigenesis. In this study, we found that Grb2 was mainly SUMOylated at K^56^, which could be removed by SENP1. It should be noted that K56 may also be modified by acetylation or ubiquitination, however we did not observe obvious obvious acetylation and ubiquitination for Grb2 (data not shown). Grb2 SUMOylation plays an important role in strengthening tumorigenesis and cell migratory capacity by enhancing the ERK activities. Moreover, our results revealed that the enhancement of ERK activities was attributed to the SUMO1 modification of Grb2 facilitating Grb2 binding with Sos1 other than other interacting proteins such as EGFR, SHP2, FAK, PTPα and Gab1, which are all considered connecting the ERK activities and cell phenotypes.

As the activator of Ras/MEK/ERK pathway, Grb2-Sos1 complex is crucial for regulating cell proliferation, tumorigenesis, early embryonic stem cell fate and normal development [35]. Grb2 and Sos1 function interdependently on each other, thus, mutations on Sos1 or unusual phosphorylation on Grb2 would lead to aberrant physiological events such as Noonan syndrome [36,37] and tumorigenesis [11]. Hence, the anti-cancer strategies targeting Grb2 is also focused on the disruption of the Grb2-Sos1 complex by blocking the interaction of Grb2 SH3 domain with Sos1 [38,39]. In this study, we discovered the formation of Grb2-Sos1 complex is finely regulated by SUMO1 modification at K^56^ of Grb2. As known, Grb2 recognizes proline-rich sequences of Sos1 by means of its SH3 domains, leading to Ras activation, whereas K^56^ of Grb2 is located in the linker region between the N-SH3 domain and the SH2 domain. The exact mechanism underlying Grb2 SUMOylation at K^56^ recruiting more Sos1 to form SUMO-Grb2-Sos1 complex deserves further study.

## Conclusions

**Figure 6:**
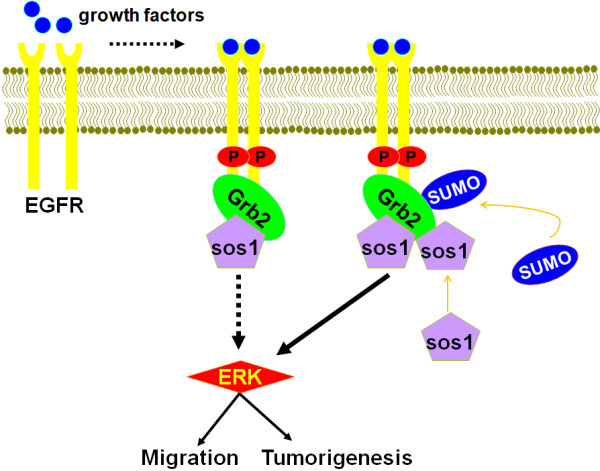
**A schematic model of Grb2 SUMOylation-regulated ERK pathway.** SUMOylation of Grb2 at K^56^ enhances the ERK activity *via* recruiting more Sos1 to form Grb2-Sos1 complex, and consequently promoting cell migration and tumorigenesis. Dashed line----Weak; Solid line----Strong.

In summary, as shown in Figure 6, our data unravel a new regulatory mechanism that Grb2 SUMOylation plays an important role in enhancing the Ras/MEK/ERK pathway. Grb2 SUMOylation at K^56^ facilitates the formation of Grb2-Sos1 complex *via* recruiting more Sos1, leading to the ERK activation and consequently promoting cell migration and tumorigenesis. Our findings might also have significant impact on cancer therapy, as dysregulation of the Ras/MEK/ERK pathway is associated with diverse cancers.

## Methods

### Antibodies and reagents

Antibodies against Grb2 (#3972), Sos1 (#5890), SUMO-1 (#4930), Phospho-P44/42-Erk1/2 (#4370), P44/42-Erk1/2 (137F5), β-Actin (13E5) were from Cell signaling Technology. GAPDH (ab37168) antibody was from ABCAM. Normal mouse IgG antibody (sc-2025) was from Santa Cruz Biotechnology (Santa Cruz, CA). Phospho-SHP2 pY542 (#2184-1), phospho-EGFR pY1068 (#1138-1) antibodies were from EPITOMICS. Anti-Flag (M2) and anti-HA mouse antibodies were from Sigma. Protein G Plus/Protein A agarose suspension (#IP05) and Recombinant Human Epidermal Growth Factor (rHu EGF) were purchased from Calbiochem. Doxycycline Hyclate (D9891) and Puromycin (P8833) were from Sigma.

### Plasmids

The Grb2 cDNA was cloned and sequenced, and then sub-cloned into the vector pCMV-Tag2b. The Flag-Grb2 was cloned into the lentiviral vector carrying *Puromycin* and *EGFP* genes [13]. The Grb2 cDNA was cloned into the vector pGEX4T1 to generate a construct GST-Grb2. The shRNA sequence targeting mouse Grb2 3′UTR (shGrb2) was obtained from Sigma ‘Mission shRNA’ online: **5′-GCATGATGTTTAAGGCCACAT-3′** at 1270 site. The shRNA was cloned into pLKO.1 vector. The FAK cDNA was cloned into the vector pCMV-HA to generate the HA tagged FAK. The pE1E2S1 plasmid was obtained from Dr. Jiemin Wong in East China Normal University.

### Cell cultures

HEK293T, 293FT, NIH/3T3 and CMT-93 cell lines were cultured in Dulbecco’s modified Eagle’s medium (DMEM) containing 10% fetal calf serum (Hyclone) at 37°C in a 5% CO2 humidified incubator. Cell transfection was performed using Lipofectamine 2000 (Invitrogen).

### SUMOylation assays

Grb2 SUMOylation was analysed in HEK293T by the method of *in vivo* SUMOylation assay using Ni^2+^-NTA beads as previously described [12,19]. *In vitro E.coli* BL21-based SUMOylation assay with the plasmid pE1E2S1 was conducted as previously described [14].

### Soft agar colony assay

The effect of Grb2^WT^ and Grb2^K56R^ on cellular transformation and tumorigensis was assessed by using a soft agar colony assay as previously described [13]. Briefly, this assay was performed in 6-well plates with a base of 2 ml of medium containing 5% (for NIH/3T3 stable cell lines) or 20% (for CMT-93 stable cell lines FBS) with 0.6% Bacto agar (Amresco). Cells were seeded in 2 ml of medium containing 5% or 20% FBS with 0.35% agar at 5 × 10^4^ (for NIH/3T3) or 5 × 10^3^ (for CMT-93) cells/well and layered onto the base. The photographs of the cells growing in the plate and of the colonies developed in soft agar were taken at day 21 (for NIH/3T3) or 14 (for CMT-93). Three independent experiments were performed in triplicate.

### Vasculogenic mimicry

The vasculogenic mimicry experiment of stable NIH/3T3 cell lines was carried out usingμ-Slide Angiogenesis Kit (IBIDI) according to the manufacturer’s protocol. Cells at a density of 1 × 10^4^ were plated in each well which was coated with matrigel and pictures were taken with microscope 20 hours later.

### Migration assay by wound healing

This method for analysis of migration was conducted as described previously [19]. Briefly, 1 × 10^4^ (for CMT-93) or 2 × 10^4^ (for NIH3T3) of cells were plated into the μ-Dish (35 mm high, purchased from IBIDI ) and cultured overnight to ensure adhered. A clear area was created by removing the Culture-Insert from the μ-Dish, and photos were taken as indicated time until the wound was healed.

### Migration assay by RTCA-DP

Migration assay by using the xCELLigence RTCA-DP system (Roche, Mannheim, Germany) was described in our previous study [20]. Briefly, 5 × 10^3^ of serum-starved stable CMT-93 cell lines were resuspended in 100 μl of serum free medium and added into the pre-equilibrated upper chamber of the CIM-plate along with the bottom-well plate containing 2% FBS DMEM medium for migration.

### Xenografted tumor model in vivo

Stable CMT-93 cell lines (2 × 10^6^, suspended in 100 sterile PBS) were harvested and injected subcutaneously into 5-week-old male BALB/c nude mice (n = 5) individually. Two weeks after injection, the tumors were measured every 3 days. At the experimental endpoint, mice were sacrificed and the tumors were dissected, weighed and photographed.

### Immunoprecipitation

Cells transfected with the indicated plasmids were lysed in the RIPA buffer (50 mM Tris–HCl pH7.4, 150 mM NaCl, 1% NP-40, 20 mM N-ethylmaleimide, and complete protease inhibitor cocktail tablet) on ice. About 500–1000 μg cell lysates were incubated with 30 μl of Protein A/G agarose and Flag or HA antibodies overnight at 4°C on an verticle roller. The next day, the Plus A/G beads were washed 4 times with RIPA buffer and dissolved in 1× protein loading buffer (50 mM Tris–HCl pH6.8, 2% SDS, 0.1% Bromophenol Blue, 10% Glycerol, 1.5% DTT) for Western Blot analysis.

### Western blot

The proteins either from whole cell lysates in the SDS-lysis buffer (10 mM Tris–HCl, pH 7.4, 1% SDS, and 1 mM Na3VO4), or derived from immunoprecipitations, were resolved via SDS/PAGE gels then transferred to a polyvinylidene difluoride (PVDF) membrane. The membrane was subsequently probed with the indicated primary antibodies and second antibodies indicated, and then exposured in ImageQuant LAS 4000 (GE) after incubating with ECL substrate.

## Abbreviations

SUMO: Small ubiquitin-related modifier; SH2: Src-homology 2 domain; SH3: Src-homology 3 domain; SENP1: Sentrin-specific protease 1; Aos1/Uba2: SUMO-activating enzyme E1; Ubc9: SUMO-conjugating enzyme; GST: Glutathione S transferase; EGFR: Epidermal growth factor receptor; shRNA: Short hairpin RNA; shGrb2: Short hairpin RNA targeting Grb2; 3′UTR: 3′ untranslated region; RTK: Receptor tyrosine kinases.

## Competing interests

The authors declare that they have no competing interests.

## Authors’ contributions

YQ performed most of the experiments; QC, JH and JY analyzed data; QC, XL, CZ, CC, XZ, RD, MX, HY and YW helped with all experiments and contributed to implementation and interpretation of the results; JH outlined the experimental design of the study, contributed to implementation and the discussion of the results; JY contributed intellectually toward the design and wrote the manuscript. All authors read and approved the final manuscript.

## Supplementary Material

Additional file 1 **(A) More representive images from the colony formation experiments (Figure **4**H) were shown. (B)** The number of colonies was counted. The colony sizes but not colony numbers were different among indicated cell lines. Click here for file

Additional file 2 Schematic representation of the binding between Grb2 and 6 selected target proteins, including EGFR, SHP2, FAK, Sos1, PTPα and Gab1. Click here for file
