# Supplementary figures and images for: SUMOylation of Grb2 enhances the ERK activity by increasing its binding with Sos1
Source: Mol Cancer. 2014 Apr 29;13:95. doi: 10.1186/1476-4598-13-95 (PMC4021559; doi:10.1186/1476-4598-13-95)

A

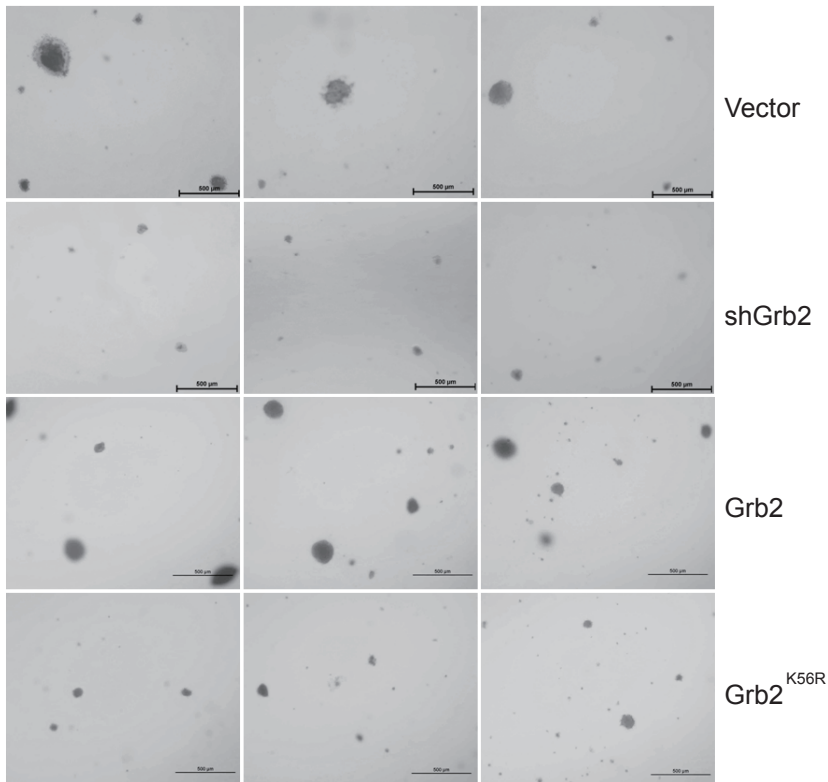

B

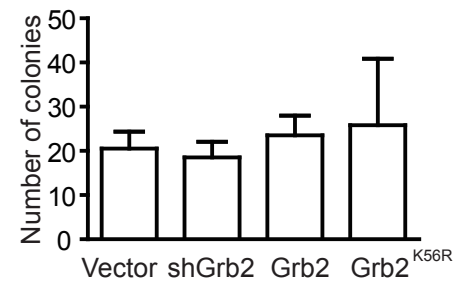

Supplement: Additional file 1 — (A) More representive images from the colony formation experiments (Figure 4H) were shown. (B) The number of colonies was counted. The colony sizes but not colony numbers were different among indicated cell lines. [file 1476-4598-13-95-S1.pdf]

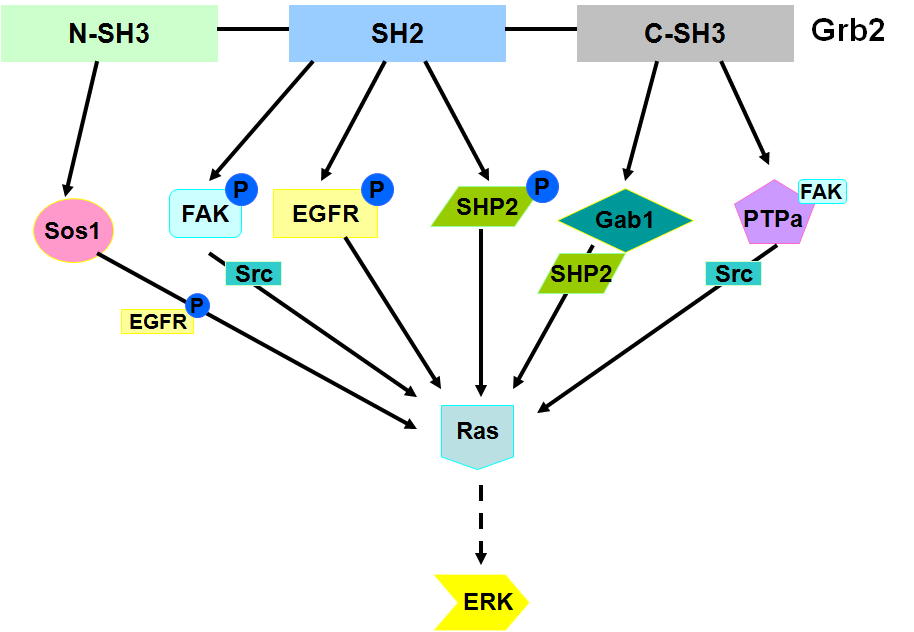

Supplement: Additional file 2 — Schematic representation of the binding between Grb2 and 6 selected target proteins, including EGFR, SHP2, FAK, Sos1, PTPα and Gab1. [file 1476-4598-13-95-S2.tiff]
